# The contribution of IL-6 to beta 3 adrenergic receptor mediated adipose tissue remodeling

**DOI:** 10.14814/phy2.12312

**Published:** 2015-02-23

**Authors:** Samyra L Buzelle, Rebecca E K MacPherson, Willem T Peppler, Laura Castellani, David C Wright

**Affiliations:** 1 Department of Biochemistry, Ribeirao Preto Medical School, University of Sao PauloRiberao Preto, Brazil; 2 Department of Human Health and Nutritional Sciences, University of GuelphGuelph, Ontario, Canada

**Keywords:** Adipose tissue, beta 3 adrenergic receptor, IL-6, mouse

## Abstract

The chronic activation of beta 3 adrenergic receptors results in marked alterations in adipose tissue morphology and metabolism, including increases in mitochondrial content and the expression of enzymes involved in lipogenesis and glyceroneogenesis. Acute treatment with CL 316,243, a beta 3 adrenergic agonist, induces the expression of interleukin 6. Interestingly, IL-6 has been shown to induce mitochondrial genes in cultured adipocytes. Therefore, the purpose of this paper was to examine the role of interleukin 6 in mediating the in vivo effects of CL 316,243 in white adipose tissue. Circulating IL-6, and markers of IL-6 signaling in white adipose tissue were increased 4 h following a single injection of CL 316,243 in C57BL6/J mice. Once daily injections of CL 316,243 for 5 days increased the protein content of a number of mitochondrial proteins including CORE1, Cytochrome C, PDH, MCAD, and Citrate Synthase to a similar extent in adipose tissue from WT and IL-6^−/−^ mice. Conversely, CL 316,243-induced increases in COXIV and phosphorylated AMPK were attenuated in IL-6^−/−^ mice. Likewise, the slight, but significant, CL 316,243-induced increases in ATGL, PEPCK, and PPAR*γ*, were reduced or absent in adipose tissue IL-6^−/−^ mice. The attenuated response to CL 316,243 in white adipose tissue in IL-6^−/−^ mice was associated with reductions in whole-body oxygen consumption and energy expenditure in the light phase. Our findings suggest that IL-6 plays a limited role in CL 316,243-mediated adipose tissue remodeling.

## Introduction

Beta 3 adrenergic receptors are expressed almost exclusively in adipocytes (Granneman et al. 1991) and their targeted activation has been shown to have profound effects on adipose tissue morphology and metabolism. The chronic, in vivo activation of beta 3 adrenergic receptors with the potent and highly selective agonist CL 316,243 (CL) (Bloom et al. 1992) results in reductions in unilocular fat cell size and increases in the appearance of multilocular fat cells in white adipose tissue depots (Granneman et al. 2005). In addition, prolonged treatment (i.e., 3–7 days) with CL increases mitochondrial proteins (Mottillo et al. 2007, 2014), including Uncoupling Protein 1 (UCP-1), in parallel with the induction of enzymes involved in de novo lipogenesis and glyceroneogenesis (Mottillo et al. 2014). These changes in adipose tissue morphology and metabolism are associated with increases in whole-body oxygen consumption (Granneman et al. 2003).

The activation of beta 3 adrenergic receptors by CL leads to rapid and robust increases in fatty acid release and this is associated with increases in the expression of IL-6 (interleukin 6), and the induction of SOCS3 (Suppressor of Cytokine Signaling 3), a transcriptional target of IL-6 signaling, in white adipose tissue (Castellani et al. 2014). The induction of IL-6 by CL is likely the result of increases in fatty acids as CL-induced increases in proinflammatory cytokines are attenuated when the release of fatty acids is blocked (Mottillo et al. 2010). Mounting evidence would suggest that IL-6 could play a role in mediating the effects of CL on adipose tissue mitochondrial proteins. First, we have reported that in cultured adipose tissue IL-6 can directly induce the expression of mitochondrial genes such as, COXIV (Cytochrome C Oxidase Subunit IV) and CPT-1 (Carnitine Palmitoyl Transferase 1) (Wan et al. 2012a). Second, exercise training and cold stress-induced increases in UCP-1 are attenuated in adipose tissue from IL-6^−/−^ mice (Knudsen et al. 2014). Third, treating adipocytes (Kelly et al. 2004) or cultured adipose tissue (Wan et al. 2012b) with IL-6 activates 5′AMP activated protein kinase (AMPK), an enzyme previously shown to control the expression of mitochondrial proteins in adipose tissue (Wan et al. 2014). Similarly, long term in vivo CL treatment activates AMPK in white adipose tissue (Mulligan et al. 2007). However, it is unclear if the increase in AMPK activity, similar to what has been shown in brown adipose tissue (BAT) after cold exposure (Mulligan et al. 2007), is related to increases in AMPK protein content. Collectively, these findings provide evidence that IL-6 could be involved in mediating the effects of CL on adipose tissue mitochondrial and perhaps lipogenic and glyceroneogenic proteins. To the best of our knowledge this has not been explored. Within this context the purpose of the current investigation was to examine the role of IL-6 in mediating the response of adipose tissue to CL. We hypothesized that CL-mediated increases in adipose tissue mitochondrial, glyceroneogenic and lipogenic proteins would be attenuated in IL-6^−/−^ mice.

## Materials

Reagents, molecular weight marker, and nitrocellulose membranes for SDS-PAGE were purchased from Bio-Rad (Mississauga, ON). Antibodies against AMPK alpha (#2532), phospho AMPK^Thr172^ alpha (#2535), STAT3 (Signal Transducer and Activator of Transcription 3) (#8768), phospho STAT3^Tyr705^ (#9138), Acetyl CoA Carboxylase (ACC), (#3676), Fatty Acid Synthase (FAS) (#3180) Adipose Tissue Triglyceride Lipase (ATGL) (#2439), and Peroxisome Proliferator Activated Receptor gamma (PPAR*γ*)(#2430) were from Cell Signaling (Danvers, MA). Antibodies for Citrate Synthase (CS), (# ab129095) and COX IV (Cytochrome C Oxidase Subunit IV) (# ab16056) were from Abcam (Cambridge, MA). Antibodies against the E1 alpha subunit of pyruvate dehydrogenase (PDH) (# MSP03), Cytochrome C (#MSA06), and CORE1 (Complex III, subunit CORE1) (#MS303) were purchased from Mitosciences (Eugene, OR). Antibodies against Phosphoenolpyruvate carboxykinase (PEPCK) (#10004943) and Medium-chain fatty acyl-CoA dehydrogenase (MCAD) (#101730) were purchased from Cayman Chemical (Ann Arbor, MI). ECL Plus was a product of Amersham Pharmacia Biotech (Arlington Heights, IL). SuperScript II Reverse Transcriptase, oligo(dT) and dNTP were from Invitrogen (Burlington, ON). Taqman Gene Expression Assays for mouse *β* actin (4352933_0911029), UCP-1 (# Mm01244861_m1), IL-6 (# Mm00446190_m1), SOCS3 (# Mm00545913_s1) and PGC-1α (PPAR*γ* co-activator 1 alpha) (# Mm01208835_m1) were from Applied Biosytems (Foster City, CA). All other chemicals were purchased from Sigma (Oakville, ON).

### Animals

Eight-week-old male wild-type (WT, C57BL6/J) and IL-6^−/−^ (B6.129S2-Il6 < tm1Kopf>/J) mice were purchased from Jackson Laboratory (Bar Harbor, ME). As described previously (O'Neill et al. 2013a), the control mice used in this study were recommended by the distributor as they provided the C57BL/6J background used to backcross IL-6 KO 129S mice for 11 generations (Kopf et al. 1994). Moreover, the IL-6 KO mice used in this study are crossed with a pure C57BL/6J line every 10 generations to prevent genetic drift. Mice were housed in individual cages, with a 12:12-h light-dark cycle with ad libitum access to water and standard rodent chow Over the 5 days of the study, body mass, and food intake were recorded daily. All experimental procedures were approved by the University of Guelph Animal Care Committee and followed Canadian Council on Animal Care guidelines.

#### Acute CL treatment

Male C57BL6/J mice were injected I.P. with CL (1.0 mg/kg bw) or an equivalent volume of sterile saline. 4 h postinjection mice were anesthetized with sodium pentobarbital (5 mg/100 g bw I.P.) and epididymal adipose tissue (eWAT), inguinal adipose tissue (iWAT), skeletal muscle (triceps), and liver harvested. Tissues were cleaned of visible blood, snap frozen in liquid nitrogen and stored at −80°C until further analysis.

#### Five day CL treatment

Wild-type and IL-6^−/−^ mice were injected (I.P.) with either CL (1.0 mg/kg bw) or an equivalent volume of sterile saline once per day, for five consecutive days. Injections occurred at ∼8:00 AM in the morning which occurred at the beginning of the animals light cycle.

#### Metabolic caging

Immediately following the final injection of either saline or CL, mice were placed in a Comprehensive Lab Animal Monitoring System (CLAMS, Columbus Instruments, Columbus, OH) for the determination of oxygen consumption, carbon dioxide production, respiratory exchange ratio (RER), and heat production. The acute effects of CL on energy expenditure and oxygen consumption are transient and return to control levels 8 h following treatment (Lateef et al. 2014). Thus, we excluded the initial 8-h period from our data analysis to focus on the chronic effects of CL on whole-body fuel utilization. The light cycle data were averaged per animal from 16:00–20:00 on day 1, and 08:00–10:00 on day 2. The dark cycle data were calculated from 20:00–08:00. Animals were provided with water and standard rodent chow throughout the CLAMS experiment.

#### Glucose tolerance tests

Approximately 30 h following the last CL injection mice underwent an intraperitoneal glucose tolerance test (GTT). For this, mice were fasted for 6 h prior to an I.P. injection of glucose (2 g/kg body weight) and blood glucose was assessed over 120 min by tail vein sampling at the indicated intervals using a glucometer (Freestyle Lite, Abbott Diabetes Care Inc., Alameda, CA). Changes in glucose over time were plotted, and the area under the curve (AUC) was calculated for each. Following the GTT, mice were anesthetized with sodium pentobarbital and adipose tissue (eWAT, iWAT, BAT) was, removed, weighed, and snap frozen in liquid nitrogen and stored at −80°C.

### Western blotting

Tissue samples were homogenized in ice-cold NP-40 cell lysis buffer (Life Technologies), supplemented with phenylmethylsulfonyl fluoride and protease inhibitor cocktail (Sigma) in a dilution of 3× for WAT and 6× for BAT. Homogenized samples were centrifuged at 4°C (10 min at 5000 *g*), the infranatant was collected, and protein content determined using a bicinchoninic acid assay (Smith et al. 1985). Equal amounts of protein were separated on SDS-PAGE gels, and transferred onto nitrocellulose membranes. The membranes were stained with Ponceau S to ensure equal loading and were blocked with 5% powdered nonfat milk in TBST for 1 h and then incubated at 4°C overnight with primary antibodies. The following day, membranes were incubated with appropriate horseradish peroxidase-conjugated donkey anti-rabbit or goat anti-mouse IgG secondary antibodies (Jackson Immuno-Research Laboratories) diluted in TBST/1% nonfat dry milk for 1 h at room temperature. Signals were detected using enhanced chemiluminescence and were subsequently quantified by densitometry using a FluorChem HD imaging system (Alpha Innotech, Santa Clara, CA).

### Plasma IL-6

Changes in circulating IL-6 were measured using mouse specific ELISA as described in detail previously (Castellani et al. 2014).

### Real-time PCR

Changes in IL-6, SOCS3, and UCP-1 mRNA expression were determined using real-time qPCR as described previously (Frier et al. 2011; Wan et al. 2012b). RNA was isolated using an RNeasy kit according to the manufacturer's instructions. Reverse transcription of RNA to complementary DNA (cDNA) was performed using 1 *μ*g of RNA, SuperScript II Reverse Transcriptase, dNTP, and oligo(dT). Real-time PCR was completed using a 7500 Fast Real-Time PCR system (Applied Biosystems, Foster City, CA). Samples were run in triplicate. Each well contained 1 *μ*L gene expression reagents, 1 *μ*L cDNA template, 10 *μ*L Taqman Fast Universal PCR Master Mix, and 8 *μ*L RNase-free water. Changes in mRNA expression were expressed relative to the expression of *β* actin, and differences in gene expression were determined using the 2^−ΔΔCT^ method (Livak and Schmittgen 2001).

### Histochemistry

Epididymal and inguinal adipose tissue samples were fixed in 10% neutral-buffered formalin and then processed for hematoxylin and eosin staining as we have described previously (Beaudoin et al. 2013). The percentage of multilocular adipocytes was expressed as a percentage of adipocytes examined (Himms-Hagen et al. 2000). Unilocular and multilocular adipocytes were classified when one, or more than one, lipid droplet were identified within the plasma membrane, respectively. Images were obtained at 40× magnification, and counting of adipocytes was completed across an entire field by a blinded observer. Approximately 500 cells per animal were analyzed using ImageJ Software (http://rsbweb.nih.gov/ij/).

### Statistical analysis

Data are reported as means ± standard error (SE). Data were analyzed using a two-way analysis of variance (ANOVA) with LSD post hoc analysis to determine differences between groups. The fold change between WT and IL-6^−/−^ group was analyzed by Student's *t*-test. Total area under the curve (AUC) for GTT was calculated using the trapezoid method (GraphPad Prism 5, La Jolla, CA). Statistical significance was set at *P* < 0.05.

## Results

### Markers of IL-6 signaling are increased in white adipose tissue following acute CL treatment

**Figure 1:**
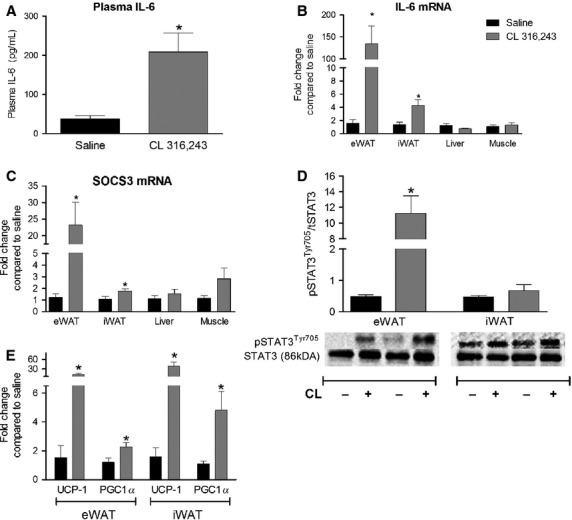
A single injection of CL 316,243 increases indices of IL-6 signaling in white adipose tissue. Male C57BL6/J mice were injected with CL (1.0 mg/kg bw) or an equivalent volume of sterile saline (sal) and epididymal adipose tissue (eWAT), inguinal adipose tissue (iWAT), liver, skeletal muscle (triceps) and plasma harvested 4 h following injections for the determination of changes in the (A) circulating IL-6, the mRNA expression of (B) IL-6, (C) SOCS3, (E) PGC-1α/UCP-1 and (D) the phosphorylation of STAT3. Data are presented as mean + SEM for 5–9 mice/group and are expressed relative to the saline-treated control group. Representative blots are shown below the quantified data in C.

To elucidate the role of IL-6 in mediating the effects of CL in adipose, we first wanted to demonstrate the activation of IL-6 signaling in white adipose tissue following acute CL treatment. Four hours following a bolus injection of CL (1.0 mg/kg bw) in male C57BL6/J mice, circulating IL-6 levels were increased (Fig.1A). IL-6 (Fig.1B) and SOCS3 (Fig.1C) expression were increased in both eWAT and iWAT. The expression of these genes were not increased in skeletal muscle (triceps) or liver. The phosphorylation of STAT3, a component of the Janus Kinase (JAK)-Signal Transducer and Activator of Transcription (STAT) signaling pathway that is activated by IL-6 (Babon and Nicola 2012), was increased in eWAT, but not iWAT (Fig.1D). CL treatment increased the mRNA expression of PGC-1α, a regulator of mitochondrial biogenesis (Kleiner et al. 2012), and UCP-1 in both eWAT and iWAT (Fig.1E). These findings provide evidence that CL increases indices of IL-6 signaling in mouse white adipose tissue in vivo, that this occurs to a greater extent in eWAT compared to iWAT, and is associated with the induction of mitochondrial genes and transcriptional coactivators in white adipose tissue.

### CL-induced changes in body weight and adipose tissue

**Figure 2:**
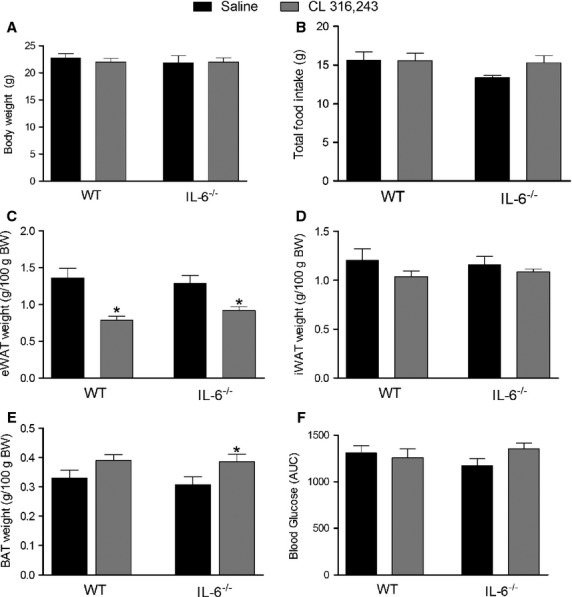
Treatment with CL 316,243 reduces epididymal adipose tissue weight independent of changes in body weight, food intake, and glucose tolerance. WT and whole-body IL-6^−/−^ mice were injected with CL (1.0 mg/kg bw) or an equivalent volume of sterile saline once per day for five consecutive days and (A) final body weight, (B) total food intake, (C) eWAT weight, (D) iWAT weight, (E) BAT weight and (F) the area under the curve (AUC) during a glucose tolerance test determined. Data are presented as mean + SEM for seven mice/group. *Significantly different than saline-treated group in the same genotype, *P* < 0.05.

Having demonstrated that CL increases IL-6 signaling in white adipose tissue we next sought to determine if IL-6 was required for CL-induced changes in mitochondrial, glyceroneogenic and lipogenic proteins. To test this we treated WT and IL-6^−/−^ mice with CL (1.0 mg/kg bw, IP injection) or an equivalent volume of sterile saline, once per day for 5 days. This duration of CL treatment has previously been shown to increase adipose tissue mitochondrial proteins (Mottillo et al. 2007). There were no differences in final body weight (Fig.2A) or cumulative food intake (Fig.2B) between WT and IL-6^−/−^ mice treated with saline or CL. Five days of CL treatment led to reductions in eWAT (Fig.2C), but not iWAT (Fig.2D) fat pad weight. The CL-mediated reduction in epididymal adipose tissue was greater in WT compared to IL-6^−/−^ mice (42.4% decrease compared to WT saline 29.4% decrease compared to IL-6^−/−^ saline *P* < 0.05). CL treatment increased intrascapular brown adipose tissue mass in IL-6^−/−^ but not in WT mice (Fig.2E).

As changes in glucose homeostasis have been linked to alterations in adipose tissue mitochondrial proteins (Sutherland et al. 2008) we thought it necessary to determine if CL treatment altered glucose homeostasis in WT and IL-6^−/−^ mice. Despite reducing fat pad mass CL treatment did not change glucose tolerance (Fig.2F).

**Figure 3:**
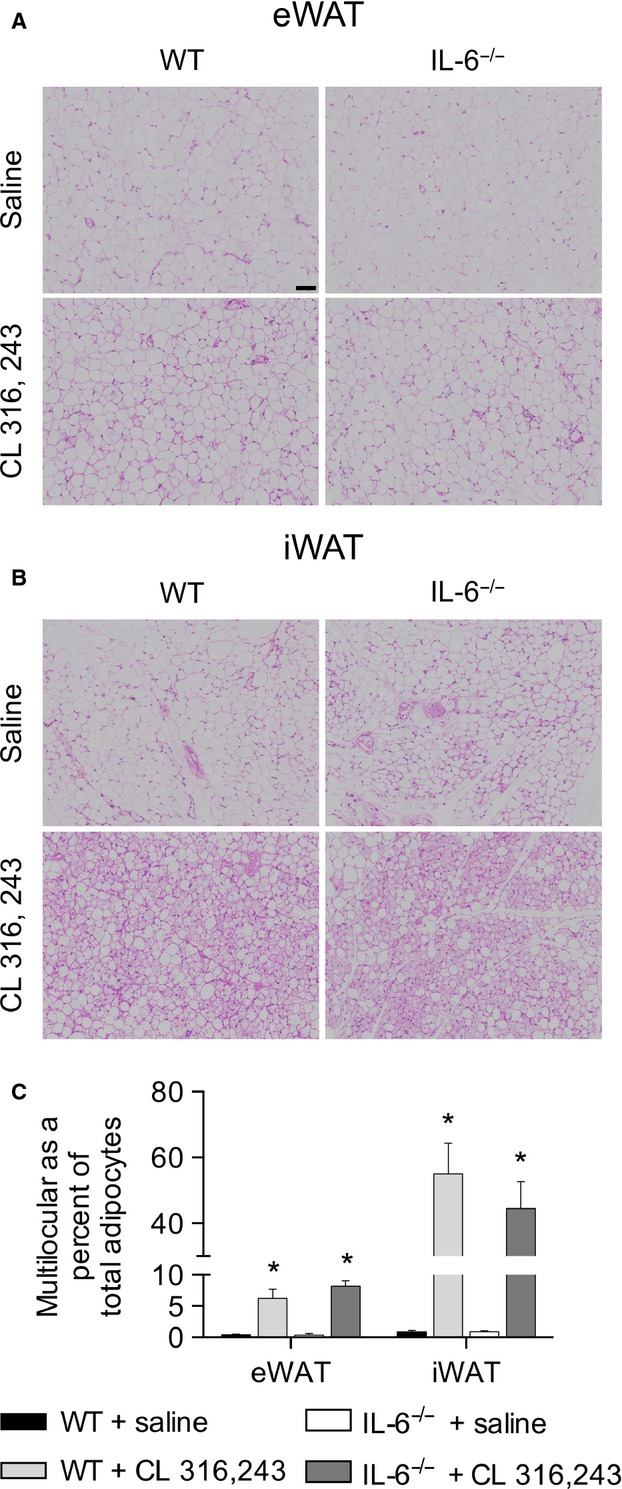
Treatment with CL 316,243 increases the appearance of multilocular fat cells in epididymal (A) and inguinal (B) adipose tissue from WT and IL-6^−/−^ mice. Adipose tissue sections were stained with hematoxylin and eosin and imaged under 40× magnification. Approximately 500 cells per animal was analyzed and the ratio of multilocular to total adipocytes was calculated for eWAT (C) and (D) iWAT. The black scale bar is 71 *μ*m.

The chronic activation of beta 3 adrenergic receptors leads to the appearance of multilocular fat cells (Granneman et al. 2005). To determine if IL-6 was required for this we histologically examined eWAT and iWAT from saline and CL-treated WT and IL-6^−/−^ mice. CL treatment resulted in robust increases in the appearance of multilocular fat cells in eWAT (Fig.3A, C) and iWAT (Fig.3B, D) in both genotypes.

### CL-induced changes in whole-body energy expenditure

**Figure 4:**
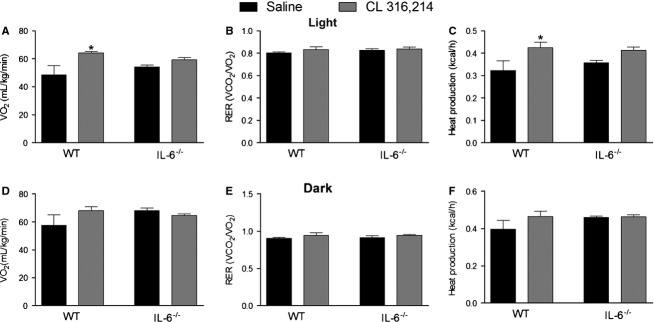
CL 316,243 treatment increases oxygen consumption and heat production in WT but not IL-6^−/−^ mice. WT and whole-body IL-6^−/−^ mice were injected with CL (1.0 mg/kg bw) or an equivalent volume of sterile saline once per day for five consecutive days and oxygen consumption (A, D), respiratory exchange ratio (RER) (B, E) and heat production (C, F) measured in metabolic caging in the light (A–C) and dark (D–F) cycles. Data are presented as mean + SEM for four mice/group. *Significantly different than saline-treated group in the same genotype, *P* < 0.05.

Longer term CL treatment has been reported to increase whole-body oxygen consumption in mice (Granneman et al. 2003). In the current study CL treatment increased oxygen consumption (Fig.4A) and energy expenditure (Fig.4C) in WT but not IL-6^−/−^ mice in the light cycle, whereas RER was not different between groups (Fig.4B). In the dark cycle, oxygen consumption (Fig.4D), RER (Fig.4E) and energy expenditure (Fig.4F) were not different between groups.

### CL-induced increases in COXIV, and AMPK are attenuated in white adipose tissue from IL-6^−/−^ mice

**Figure 5:**
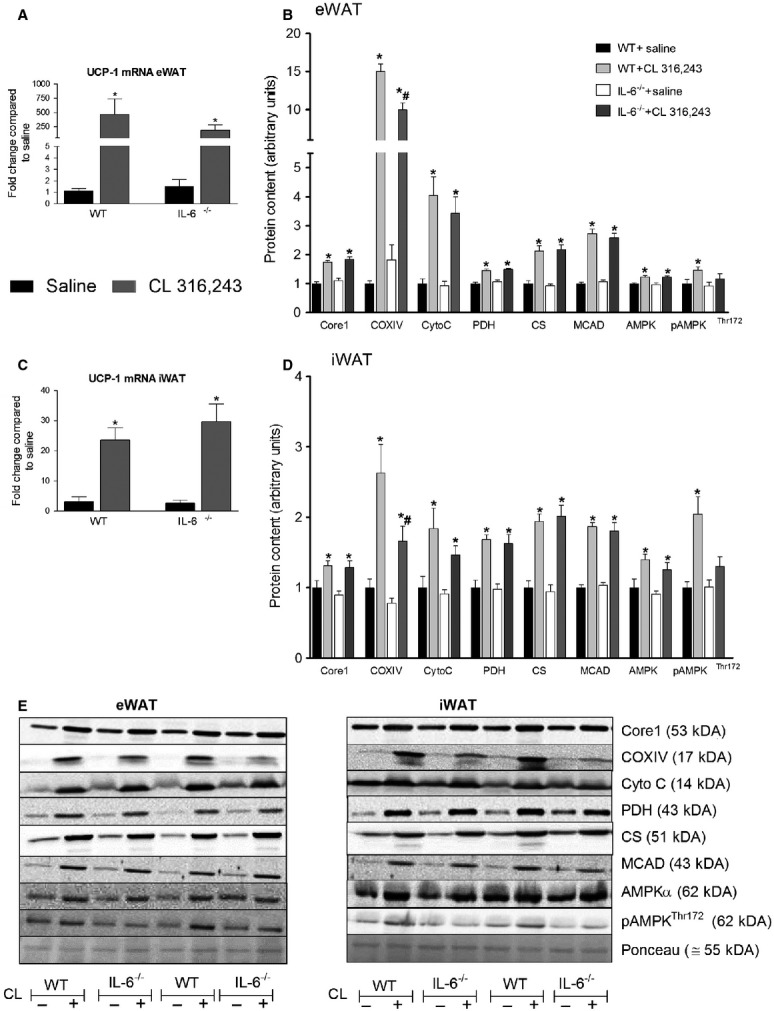
Treatment with CL 316,243 increases select mitochondrial proteins in white adipose tissue in an IL-6-dependent manner. WT and whole-body IL-6^−/−^ mice were injected with CL (1.0 mg/kg bw) or an equivalent volume of sterile saline once per day for five consecutive days and changes in UCP-1 mRNA expression (A, C) and mitochondrial proteins and AMPK (B, D) determined in epididymal adipose tissue (eWAT) (A, B) and inguinal adipose tissue (iWAT) (C, D). Data are presented as mean + SEM for seven samples/group. Representative blots are shown in E. *Significantly different than saline treated in the same genotype, #Significantly different than CL treated in WT, *P* < 0.05. CS, citrate synthase; Cyto C, cytochrome C.

To assess the role of IL-6 in mediating the effects of CL on mitochondrial biogenesis we measured changes in a number of mitochondrial enzymes in adipose tissue from WT and IL-6^−/−^ mice treated with CL for 5 days. As shown in Fig.5A, CL treatment increased UCP-1 mRNA expression ∼400 fold and 200 fold in eWAT from WT and IL-6^−/−^ mice, respectively (We were unable to consistently detect UCP-1 protein content in eWAT). CL-induced increases in the protein content of CORE1, Cytochrome C, PDH, citrate synthase, and MCAD were similar in eWAT from WT and IL-6^−/−^ mice. Conversely, the induction of COXIV protein content was attenuated in IL-6^−/−^ mice (Fig.5B). Given the reputed role of AMPK as a regulator of mitochondrial biogenesis we assessed changes in the protein content and phosphorylation of AMPK*α*. CL treatment increased the protein content of AMPK*α* in both genotypes, while the phosphorylation of AMPK*α* was only increased in WT mice treated with CL (Fig.5B).

In iWAT UCP-1 mRNA expression (Fig.5C) was increased to a similar extent in both genotypes following 5 days of CL treatment. The protein content of UCP-1 was increased to a similar extent in iWAT from both WT (1.00 ± 0.27 saline, 3.49 ± 0.21 CL *P* < 0.05) and IL-6^−/−^ (0.75 ± 0.17 saline, 2.86 ± 0.14 CL *P* < 0.05) mice. COXIV protein content was greater in iWAT from CL-treated WT compared to IL-6^−/−^ mice. The protein content of MCAD, citrate synthase, cytochrome C, CORE1, and PDH were increased with CL treatment with no differences observed between WT and IL-6^−/−^ mice. Similar to what we observed in eWAT, the protein content of AMPKα was increased in both genotypes following CL treatment whereas the phosphorylation of AMPK on threonine 172 was only increased in iWAT from WT mice.

In addition to increasing mitochondrial proteins in white adipose tissue, it has also been reported that CL treatment induces mitochondrial biogenesis in brown adipose tissue (BAT) (Mottillo et al. 2014). In contrast, in the current study CL treatment did not increase the protein content of UCP-1, citrate synthase, or COXIV (Table1).

**Table 1:** CL does not increase mitochondrial proteins in brown adipose tissue from WT or IL-6^−/−^ mice. Data are presented as means ± SEM for seven mice/group. There was no genotype or CL effect by 2 WAY ANOVA (*P* > 0.30)

	WT saline	WT CL 316,243	IL-6^−/−^ saline	IL-6^−/−^ CL 316,243
COXIV	1.00 ± 0.07	1.07 ± 0.06	1.09 ± 0.09	1.15 ± 0.05
CS	1.00 ± 0.05	1.04 ± 0.02	0.96 ± 0.03	0.92 ± 0.04
UCP-1	1.00 ± 0.08	1.13 ± 0.09	1.05 ± 0.08	1.10 ± 0.09

### CL-induced increases in lipid handling proteins are attenuated in adipose tissue from IL-6^−/−^ mice

**Figure 6:**
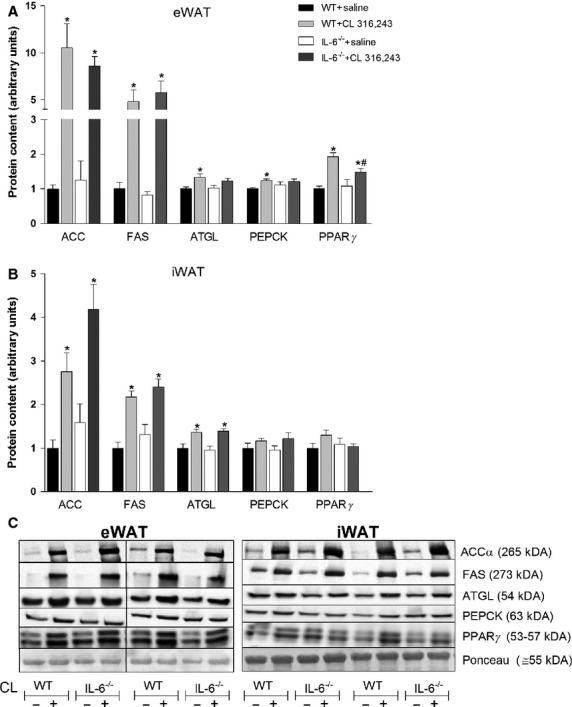
Treatment with CL 316,243 increases select lipolytic and glyceroneogenic enzymes in white adipose tissue in an IL-6-dependent manner. WT and whole-body IL-6^−/−^ mice were injected with CL (1.0 mg/kg bw) or an equivalent volume of sterile saline once per day for five consecutive days and changes in PEPCK, ATGL, FAS, ACC, and PPAR*γ* determined in epididymal adipose tissue (eWAT) (A) and inguinal adipose tissue (iWAT) (B). Data are presented as mean + SEM for seven samples/group. Representative blots are shown in C. *Significantly different than saline treated in the same genotype, ^#^significantly different than CL treated in WT, *P* < 0.05. The vertical bar in C indicates the separation of noncontiguous bands from the same gel. Please note that the PPAR*γ* antibody that was used detects both PPAR*γ*1 (lower band) and PPAR*γ*2 (upper band).

Not only does CL increase the content of mitochondrial enzymes, but it also induces the expression of enzymes involved in de novo lipogenesis and glyceroneogenesis (Mottillo et al. 2014). Therefore, it was of interest to determine if these changes were dependent on IL-6. As shown in Fig.6A, the protein content of ATGL (adipose tissue triglyceride lipase), a rate limiting enzyme of lipolysis (Haemmerle et al. 2006), was increased with CL treatment in eWAT from WT but not IL-6^−/−^ mice. Similarly, the content of PEPCK, a key enzyme regulating glyceroneogenesis (Franckhauser et al. 2002), was also increased following CL treatment in eWAT from WT, but not IL-6^−/−^ mice. The CL-mediated induction of PPAR*γ*, a transcription factor that controls the expression of ATGL and PEPCK (Tontonoz et al. 1995; Kim et al. 2006), was attenuated in IL-6^−/−^ mice. The content of FAS and ACC, enzymes that are involved in de novo lipogenesis (Ameer et al. 2014), were increased to a similar extent in eWAT from both genotypes.

In iWAT (Fig.6B) the protein content of ATGL, but not PEPCK and PPAR*γ*, was increased following CL treatment in both genotypes. FAS and ACC increased to a similar extent in inguinal adipose tissue with CL treatment in both genotypes.

## Discussion

Chronic activation of beta 3 adrenergic receptors results in a dramatic remodeling of adipose tissue that is characterized by increases in the content of mitochondrial, glyceroneogenic and lipogenic proteins (Mottillo et al. 2007, 2014). Despite robust increases in markers of IL-6 signaling in adipose tissue following an acute injection of CL the effects of longer term CL treatment on adipose tissue were largely intact in IL-6^−/−^ mice. While the majority of proteins examined did not differ between genotypes we detected small but significant differences in COXIV, p-AMPK, PEPCK, ATGL, and PPAR*γ* suggesting that the absence of IL-6 attenuates the effects of CL on the induction of these proteins.

The acute activation of beta 3 adrenergic receptors induces IL-6 mRNA expression in white adipose tissue, but not in skeletal muscle or in liver, and this coincides with increases in markers of IL-6 signaling. We interpret this as suggesting that CL-mediated increases in adipose tissue-derived IL-6, working through an autocrine/paracrine mechanism, are linked to the activation of IL-6 signaling. Of interest, CL-mediated increases in IL-6 signaling occurred to a greater extent in eWAT compared to iWAT. Previous work has demonstrated that the CL-mediated induction of proinflammatory cytokines, such as MCP-1 (Monocyte Chemoattractant Protein 1), occurs as a result of increases in adipose tissue fatty acid release (Mottillo et al. 2010). The lipolytic responsiveness of iWAT is muted compared to that of eWAT (Portillo et al. 2000; Wan et al. 2014) and thus this could potentially explain the depot specific differences in the CL-mediated activation of IL-6 signaling.

Not only does CL activate IL-6 signaling in white adipose tissue, but the induction of COXIV protein content is attenuated in adipose tissue from whole-body IL-6-deficient mice. At least in cultured adipose tissue preparations, iWAT is relatively unresponsive to IL-6 treatment (Wan et al. 2012a). In conjunction with the present results, this would suggest, at least in iWAT, that IL-6 is not sufficient to induce mitochondrial enzyme gene expression, yet is required for the effects of CL for increasing COXIV. The induction of UCP-1 has been observed in white adipose tissue beds following cold stress (Imai et al. 2006) and more recently has been shown to be induced in iWAT with exercise training (Bostrom et al. 2012; Knudsen et al. 2014). Pilegaard's group has provided data demonstrating that IL-6 plays a role in mediating the effects of exercise on inguinal UCP-1 expression (Knudsen et al. 2014). They reported that training-induced increases in UCP-1 mRNA was reduced in iWAT from IL-6^−/−^ mice (Knudsen et al. 2014). Conversely, we found that CL-mediated increases in UCP-1 mRNA expression and protein content were intact in iWAT from mice lacking IL-6. While IL-6^−/−^ mice have been widely used to examine the role of IL-6 in the response to exercise (Fritsche et al. 2010; Brandt et al. 2012; Wan et al. 2012b; O'Neill et al. 2013b; Knudsen et al. 2014; Sarvas et al. 2014) there are several caveats, particularly with regard to the effects of exercise on adipose tissue, that should be considered. For instance, plasma epinephrine levels are reduced (Tweedell et al. 2011) and the norepinephrine response to cage switch stress is attenuated in IL-6^−/−^ mice (Wernstedt et al. 2006). These findings would suggest that the attenuated induction of UCP-1 in iWAT with training could be secondary to alterations in catecholamines, a well characterized inducer of UCP-1 (Herron et al. 1990). In the present study, where beta adrenergic stimulation is being exogenously provided, this potential confounding issue would likely be avoided. In fact, we have previously shown that CL-mediated increases in plasma glycerol and fatty acid release are intact in IL-6^−/−^ mice suggesting that beta adrenergic signaling in adipose tissue is normal in these animals (MacPherson et al. 2014).

AMPK is an energy sensing enzyme and reputed regulator of mitochondrial enzymes (O'Neill et al. 2011) in a variety of tissues, including adipose (Wan et al. 2014). Similar to what has been reported following cold exposure in brown adipose tissue (Mulligan et al. 2007), the total protein content of the alpha catalytic subunit of AMPK was increased with CL treatment in both genotypes. Despite equivalent increases in AMPK*α* content the phosphorylation of the enzyme only increased following treatment in adipose tissue from WT mice. While reductions in AMPK phosphorylation mirrored the attenuation in COXIV protein content, this relationship did not hold when examining additional markers of mitochondrial biogenesis such as citrate synthase, MCAD, PDH, and CORE1. In previous work from our group we reported reductions in a number of mitochondrial proteins in eWAT, but not iWAT, from AMPK *β*1^−/−^ mice (Wan et al. 2014). However, in that particular study the attenuation of AMPK phosphorylation was much greater than in the current investigation. These results likely suggest the involvement of additional cellular mediators of adipose tissue mitochondrial enzymes.

The activation of beta 3 adrenergic receptors results in rapid and robust increases in adipose tissue lipolysis. As rates of lipolysis increase, there is a concomitant increase in fatty acid reesterfication (Vaughan 1962) and there appears to be a close relationship between lipolytic and glyceroneogenic enzymes with these proteins tracking together in mouse adipose tissue (Mennes et al. 2014). In addition, prolonged increases in lipolysis are associated with an upregulation of enzymes involved in de novo lipogenesis (Mottillo et al. 2014). Given these associations we were interested in determining if IL-6 played a role in modulating CL-induced increases in lipid handling enzymes in adipose tissue. We found that CL treatment increased the protein content of lipogenic proteins in both depots independent of IL-6. Conversely, PEPCK and ATGL protein content were modestly increased in eWAT from WT but not IL-6^−/−^ mice treated with CL and this was associated with a blunted induction of PPAR*γ*, a transcription factor that controls the expression of PEPCK and ATGL (Tontonoz et al. 1995; Kim et al. 2006). The association between reductions in ATGL and PEPCK are consistent with recent work from Mottillo et al. (2014) who reported that the inducible knockdown of ATGL prevented CL-mediated increases in PEPCK protein content. As is the case with the attenuated induction of COXIV in iWAT, the absence of a significant increase in PEPCK protein content in IL-6^−/−^ mice most likely speaks to IL-6 being required, but not sufficient, for increases in PEPCK protein content. This assumption is based in large part on previous work from our group where we demonstrated that PEPCK protein content was in fact reduced in eWAT tissue cultures following long term IL-6 treatment (Wan et al. 2012b). Differences in the models used and/or duration of treatment could also explain these differences.

Although we saw only modest phenotypic differences between WT and IL-6 KO mice with regard to adipose tissue mitochondrial and lipid handling proteins, these changes occurred in parallel with a blunted increase in whole-body oxygen consumption and heat production in the light phase. CL treatment, while not increasing mitochondrial enzymes in BAT (on a per unit protein basis), increased BAT mass but only in IL-6-deficient mice. Together these findings suggest that alterations in BAT likely do not account for the differences in whole-body oxygen consumption. In the current study the first 8 h of data collection in the metabolic caging was discarded as we wanted to avoid the acute effects of CL on energy expenditure and allow time for the mice to acclimate to the caging. While this acclimation period is longer than some have used (Wu et al. 2014), we cannot discount the possibility that the differences in oxygen consumption between groups is related to a stress response and is independent of any changes in adipose tissue metabolism.

In summary we have demonstrated that acute CL treatment leads to increases in markers of IL-6 signaling in adipose tissue in vivo and that the induction of COXIV, and the slight, but significant increases in p-AMPK, ATGL, PEPCK, and PPAR*γ* are attenuated following 5 days of CL treatment in IL-6-deficient mice. Our findings provide evidence that IL-6 could play a minor role in CL-induced adipose tissue remodeling.

## Conflict of Interest

None declared.

## References

[b1] Ameer F, Scandiuzzi L, Hasnain S, Kalbacher H, Zaidi N (2014). De novo lipogenesis in health and disease. Metabolism.

[b2] Babon JJ, Nicola NA (2012). The biology and mechanism of action of suppressor of cytokine signaling 3. Growth Factors.

[b3] Beaudoin MS, Snook LA, Arkell AM, Simpson JA, Holloway GP, Wright DC (2013). Resveratrol supplementation improves white adipose tissue function in a depot-specific manner in Zucker diabetic fatty rats. Am. J. Physiol. Regul. Integr. Comp. Physiol.

[b4] Bloom JD, Dutia MD, Johnson BD, Wissner A, Burns MG, Largis EE (1992). Disodium (R, R)-5-[2-[[2-(3-chlorophenyl)-2-hydroxyethyl]-amino] propyl]-1,3-benzodioxole-2,2-dicarboxylate (CL 316,243). A potent beta-adrenergic agonist virtually specific for beta 3 receptors. A promising antidiabetic and antiobesity agent. J. Med. Chem.

[b5] Bostrom P, Wu J, Jedrychowski MP, Korde A, Ye L, Lo JC (2012). A PGC1-alpha-dependent myokine that drives brown-fat-like development of white fat and thermogenesis. Nature.

[b6] Brandt C, Jakobsen AH, Adser H, Olesen J, Iversen N, Kristensen JM (2012). IL-6 regulates exercise and training-induced adaptations in subcutaneous adipose tissue in mice. Acta. Physiol. (Oxf).

[b7] Castellani L, Root-Mccaig J, Frendo-Cumbo S, Beaudoin MS, Wright DC (2014). Exercise training protects against an acute inflammatory insult in mouse epididymal adipose tissue. J. Appl. Physiol. (1985).

[b8] Franckhauser S, Munoz S, Pujol A, Casellas A, Riu E, Otaegui P (2002). Increased fatty acid re-esterification by PEPCK overexpression in adipose tissue leads to obesity without insulin resistance. Diabetes.

[b9] Frier BC, Jacobs RL, Wright DC (2011). Interactions between the consumption of a high-fat diet and fasting in the regulation of fatty acid oxidation enzyme gene expression: an evaluation of potential mechanisms. Am. J. Physiol. Regul. Integr. Comp. Physiol.

[b10] Fritsche L, Hoene M, Lehmann R, Ellingsgaard H, Hennige AM, Pohl AK (2010). IL-6 deficiency in mice neither impairs induction of metabolic genes in the liver nor affects blood glucose levels during fasting and moderately intense exercise. Diabetologia.

[b11] Granneman JG, Lahners KN, Chaudhry A (1991). Molecular cloning and expression of the rat beta 3-adrenergic receptor. Mol. Pharmacol.

[b12] Granneman JG, Burnazi M, Zhu Z, Schwamb LA (2003). White adipose tissue contributes to UCP1-independent thermogenesis. Am. J. Physiol. Endocrinol. Metab.

[b13] Granneman JG, Li P, Zhu Z, Lu Y (2005). Metabolic and cellular plasticity in white adipose tissue I: effects of beta3-adrenergic receptor activation. Am. J. Physiol. Endocrinol. Metab.

[b14] Haemmerle G, Lass A, Zimmermann R, Gorkiewicz G, Meyer C, Rozman J (2006). Defective lipolysis and altered energy metabolism in mice lacking adipose triglyceride lipase. Science.

[b15] Herron D, Rehnmark S, Nechad M, Loncar D, Cannon B, Nedergaard J (1990). Norepinephrine-induced synthesis of the uncoupling protein thermogenin (UCP) and its mitochondrial targeting in brown adipocytes differentiated in culture. FEBS Lett.

[b16] Himms-Hagen J, Melnyk A, Zingaretti MC, Ceresi E, Barbatelli G, Cinti S (2000). Multilocular fat cells in WAT of CL-316243-treated rats derive directly from white adipocytes. Am. J. Physiol. Cell Physiol.

[b17] Imai J, Katagiri H, Yamada T, Ishigaki Y, Ogihara T, Uno K (2006). Cold exposure suppresses serum adiponectin levels through sympathetic nerve activation in mice. Obesity (Silver Spring).

[b18] Kelly M, Keller C, Avilucea PR, Keller P, Luo Z, Xiang X (2004). AMPK activity is diminished in tissues of IL-6 knockout mice: the effect of exercise. Biochem. Biophys. Res. Commun.

[b19] Kim JY, Tillison K, Lee JH, Rearick DA, Smas CM (2006). The adipose tissue triglyceride lipase ATGL/PNPLA2 is downregulated by insulin and TNF-alpha in 3T3-L1 adipocytes and is a target for transactivation by PPARgamma. Am. J. Physiol. Endocrinol. Metab.

[b20] Kleiner S, Mepani RJ, Laznik D, Ye L, Jurczak MJ, Jornayvaz FR (2012). Development of insulin resistance in mice lacking PGC-1alpha in adipose tissues. Proc. Natl Acad. Sci. USA.

[b21] Knudsen JG, Murholm M, Carey AL, Bienso RS, Basse AL, Allen TL (2014). Role of IL-6 in exercise training- and cold-induced UCP1 expression in subcutaneous white adipose tissue. PLoS ONE.

[b22] Kopf M, Baumann H, Freer G, Freudenberg M, Lamers M, Kishimoto T (1994). Impaired immune and acute-phase responses in interleukin-6-deficient mice. Nature.

[b23] Lateef DM, Abreu-Vieira G, Xiao C, Reitman ML (2014). Regulation of body temperature and brown adipose tissue thermogenesis by bombesin receptor subtype-3. Am. J. Physiol. Endocrinol. Metab.

[b24] Livak KJ, Schmittgen TD (2001). Analysis of relative gene expression data using real-time quantitative PCR and the 2(-Delta Delta C(T)) Method. Methods.

[b25] MacPherson RE, Castellani L, Beaudoin MS, Wright DC (2014). Evidence for fatty acids mediating CL 316,243-induced reductions in blood glucose in mice. Am. J. Physiol. Endocrinol. Metab.

[b26] Mennes E, Dungan CM, Frendo-Cumbo S, Williamson DL, Wright DC (2014). Aging-associated reductions in lipolytic and mitochondrial proteins in mouse adipose tissue are not rescued by metformin treatment. J. Gerontol. A Biol. Sci. Med. Sci.

[b27] Mottillo EP, Shen XJ, Granneman JG (2007). Role of hormone-sensitive lipase in beta-adrenergic remodeling of white adipose tissue. Am. J. Physiol. Endocrinol. Metab.

[b28] Mottillo EP, Shen XJ, Granneman JG (2010). beta3-adrenergic receptor induction of adipocyte inflammation requires lipolytic activation of stress kinases p38 and JNK. Biochim. Biophys. Acta.

[b29] Mottillo EP, Balasubramanian P, Lee YH, Weng C, Kershaw EE, Granneman JG (2014). Coupling of lipolysis and de novo lipogenesis in brown, beige, and white adipose tissues during chronic beta3-adrenergic receptor activation. J. Lipid Res.

[b30] Mulligan JD, Gonzalez AA, Stewart AM, Carey HV, Saupe KW (2007). Upregulation of AMPK during cold exposure occurs via distinct mechanisms in brown and white adipose tissue of the mouse. J. Physiol.

[b31] O'Neill HM, Maarbjerg SJ, Crane JD, Jeppesen J, Jorgensen SB, Schertzer JD (2011). AMP-activated protein kinase (AMPK) beta1beta2 muscle null mice reveal an essential role for AMPK in maintaining mitochondrial content and glucose uptake during exercise. Proc. Natl Acad. Sci. USA.

[b33] O'Neill HM, Palanivel R, Wright DC, Macdonald T, Lally JS, Schertzer JD (2013b). IL-6 is not essential for exercise-induced increases in glucose uptake. J. Appl. Physiol.

[b34] Portillo MP, Villaro JM, Torres MI, Macarulla MT (2000). In vivo lipolysis in adipose tissue from two anatomical locations measured by microdialysis. Life Sci.

[b35] Sarvas JL, Niccoli S, Walser E, Khaper N, Lees SJ (2014). Interleukin-6 deficiency causes tissue-specific changes in signaling pathways in response to high-fat diet and physical activity. Physiol. Rep.

[b36] Smith PK, Krohn RI, Hermanson GT, Mallia AK, Gartner FH, Provenzano MD (1985). Measurement of protein using bicinchoninic acid. Anal. Biochem.

[b37] Sutherland LN, Capozzi LC, Turchinsky NJ, Bell RC, Wright DC (2008). Time course of high-fat diet-induced reductions in adipose tissue mitochondrial proteins: potential mechanisms and the relationship to glucose intolerance. Am. J. Physiol. Endocrinol. Metab.

[b38] Tontonoz P, Hu E, Devine J, Beale EG, Spiegelman BM (1995). PPAR gamma 2 regulates adipose expression of the phosphoenolpyruvate carboxykinase gene. Mol. Cell. Biol.

[b39] Tweedell A, Mulligan KX, Martel JE, Chueh FY, Santomango T, McGuinness OP (2011). Metabolic response to endotoxin in vivo in the conscious mouse: role of interleukin-6. Metabolism.

[b40] Vaughan M (1962). The production and release of glycerol by adipose tissue incubated in vitro. J. Biol. Chem.

[b41] Wan Z, Perry CG, Macdonald T, Chan CB, Holloway GP, Wright DC (2012a). IL-6 is not necessary for the regulation of adipose tissue mitochondrial content. PLoS ONE.

[b42] Wan Z, Ritchie I, Beaudoin MS, Castellani L, Chan CB, Wright DC (2012b). IL-6 indirectly modulates the induction of glyceroneogenic enzymes in adipose tissue during exercise. PLoS ONE.

[b43] Wan Z, Root-McCaig J, Castellani L, Kemp BE, Steinberg GR, Wright DC (2014). Evidence for the role of AMPK in regulating PGC-1 alpha expression and mitochondrial proteins in mouse epididymal adipose tissue. Obesity (Silver Spring).

[b44] Wernstedt I, Edgley A, Berndtsson A, Faldt J, Bergstrom G, Wallenius V (2006). Reduced stress- and cold-induced increase in energy expenditure in interleukin-6-deficient mice. Am. J. Physiol. Regul. Integr. Comp. Physiol.

[b45] Wu MV, Bikopoulos G, Hung S, Ceddia RB (2014). Thermogenic capacity is antagonistically regulated in classical brown and white subcutaneous fat depots by high fat diet and endurance training in rats: impact on whole-body energy expenditure. J. Biol. Chem.

